# Associations of Life’s Essential 8 With Mortality Among Individuals With Diabetes and/or Hypertension: Statistical Mediation by Inflammation and Biological Aging

**DOI:** 10.1155/mi/3674573

**Published:** 2026-05-25

**Authors:** Xixi Dong, Xuejing Zhong, Yichen Lin, Bingqin Xie, Xuefang Li, Baochang He, Fa Chen, Lingjun Yan

**Affiliations:** ^1^ Department of Epidemiology and Health Statistics, School of Public Health, Fujian Medical University, Fuzhou, China, fjmu.edu.cn; ^2^ Longyan First Affiliated Hospital of Fujian Medical University, Longyan, China, fjmu.edu.cn; ^3^ Department of Neurosurgery, Neurosurgery Research Institute, The First Affiliated Hospital, Fujian Medical University, Fuzhou, Fujian, China, fjmu.edu.cn; ^4^ Fujian Provincial Institutes of Brain Disorders and Brain Sciences, The First Affiliated Hospital, Fujian Medical University, Fuzhou, Fujian, China, fjmu.edu.cn; ^5^ Department of Neurosurgery, Binhai Branch of National Regional Medical Center, The First Affiliated Hospital, Fujian Medical University, Fuzhou, Fujian, China, fjmu.edu.cn

**Keywords:** all-cause mortality, cancer mortality, diabetes, heart disease mortality, hypertension, life’s essential 8

## Abstract

**Background:**

Despite extensive evidence supporting the American Heart Association (AHA)’s life’s essential 8 (LE8) framework for cardiovascular health (CVH) assessment, the underlying biological mechanisms linking LE8 to mortality outcomes in high‐risk populations remain unexplored. This study aimed to investigate the association between LE8 scores and mortality risk among individuals with diabetes, hypertension, and their coexistence, and explored whether inflammation and biological aging statistically mediate these relationships.

**Methods:**

We conducted a large‐scale longitudinal analysis using National Health and Nutrition Examination Survey (NHANES) data (2005–2018), including 4939 individuals with diabetes, 13,298 with hypertension, and 3303 with both conditions. LE8 scores were calculated from eight CVH metrics, with mortality ascertained through the National Death Index (NDI). Mediation analyses examined the roles of inflammation markers (neutrophil‐to‐lymphocyte ratio [NLR] and pan‐immune–inflammation value [PIV]) and phenotypic age acceleration (PhenoAgeAccel).

**Results:**

Higher LE8 scores were significantly associated with reduced all‐cause mortality and heart disease mortality across all groups (*p* < 0.001). Stratified analyses showed stronger associations among younger individuals (≤60 years) and those with higher socioeconomic status. In mediation analyses, inflammatory markers and PhenoAgeAccel statistically explained a meaningful proportion of the LE8‐mortality associations, with different patterns across disease groups. For all‐cause mortality, in diabetes, NLR and PIV accounted for larger proportions of the association (NLR: 31.6%; PIV: 26.9%), whereas in hypertension, PhenoAgeAccel accounted for a larger proportion (56.3%). Among individuals with both conditions, PhenoAgeAccel (26.1%) and NLR (5.3%) contributed to the association. Similar patterns were observed for heart disease mortality.

**Conclusion:**

Higher LE8 scores are associated with reduced mortality risk in individuals with diabetes and/or hypertension, with inflammation and biological aging statistically mediating these associations in an exploratory manner. These findings suggest potential statistical mediators that may inform future mechanistic research and therapeutic targets, but causal interpretation requires further longitudinal studies.

## 1. Introduction

Cardiovascular disease (CVD) represents a significant health challenge globally, encompassing various conditions affecting the heart and vascular system, including coronary artery disease, heart failure, and stroke. It is characterized by substantial morbidity and mortality, necessitating extensive healthcare resources and contributing significantly to the economic burden worldwide [1]. As highlighted in the Global Burden of Disease 2021, ischemic heart disease, diabetes mellitus, and stroke rank among the leading contributors to the global disease burden [2]. Despite recent declines in age‐standardized mortality rates from CVD in the U.S., the overall health and financial implications remain profound, indicating a persistent need for effective prevention strategies [3, 4].

Globally, rapidly rising rates of diabetes and hypertension significantly exacerbate the CVD epidemic. The prevalence of hypertension in the U.S. is approximately 47%, while in Europe, it is around 55% [5]. Effective identification and management of these modifiable risk factors are critical for reducing CVD morbidity and mortality. Notably, lifestyle interventions have shown promise in delaying the onset of hypertension and diabetes [6]. The International Diabetes Federation estimates that the number of individuals with diabetes in China reached approximately 140.9 million in 2021, with projections suggesting an increase to 174.4 million by 2045 [7]. The coexistence of these conditions creates a particularly high‐risk profile, with complex pathophysiological interactions that accelerate cardiovascular deterioration.

Recognizing the importance of comprehensive cardiovascular health (CVH), the American Heart Association (AHA) introduced “Life’s Simple 7” (LS7) in 2010, which outlined ideal targets across seven health behaviors [8]. In response to these challenges, the AHA expanded the CVH metrics in 2022 to include sleep health, thereby evolving LS7 into “Life’s Essential 8” (LE8). This comprehensive model encompasses four health behavior factors‐sensible diet, regular physical activity, tobacco‐free living, and sufficient sleep‐alongside four health metabolic factors‐ideal body mass index (BMI), desirable lipid levels, normal blood glucose, and optimal blood pressure (BP) [9].

Despite evidence that favorable cardiovascular metrics reduce CVD events and all‐cause mortality [10], the biological mechanisms translating improved CVH into mortality benefits remain incompletely understood. Growing evidence suggests that chronic systemic inflammation plays a crucial role in the pathogenesis of both diabetes and hypertension, contributing to vascular damage, endothelial dysfunction, and accelerated atherosclerosis [11, 12]. Inflammatory markers such as neutrophil‐to‐lymphocyte ratio (NLR) [13] and pan‐immune–inflammation value (PIV) [14] have emerged as reliable predictors of cardiovascular outcomes. Concurrently, biological aging—the progressive deterioration of physiological integrity—increasingly appears as a fundamental driver of chronic disease development. Particularly, phenotypic age acceleration (PhenoAgeAccel), which measures the discrepancy between biological and chronological age, has demonstrated strong associations with mortality risk independent of traditional risk factors [15]. Despite these advances, no studies have investigated whether inflammation and accelerated biological aging mediate the protective effects of optimal CVH in high‐risk populations.

Therefore, this study aims to systematically explore not only the associations between LE8 levels and mortality risks among individuals with diabetes, hypertension, and their coexistence but also to examine whether these relationships are statistically consistent with mediation by inflammatory processes and accelerated biological aging. These statistical mediation findings may suggest potential pathways that could be explored in future mechanistic studies and may inform risk stratification strategies, potentially transforming our approach to cardiovascular risk reduction.

## 2. Methods

### 2.1. Study Design and Population

This study utilized data derived from the U.S. National Health and Nutrition Examination Survey (NHANES), which employs a stratified, multistage probability cluster sampling design. Since its inception in 1999, NHANES has collected nationally representative samples of the noninstitutionalized U.S. population biennially, aimed at evaluating the health and nutritional status of both adults and children across the country. The survey integrates health interviews and physical measurements, encompassing sociodemographic characteristics (such as age and sex), physical examinations (including height and weight), laboratory assessments (for blood glucose and lipids), and dietary intake questionnaires. All statistical analyses were carried out using survey modules that incorporated appropriate sampling weights to address the complexities of the survey design. This comprehensive survey is organized by the National Center for Health Statistics (NCHS) under the Centers for Disease Control and Prevention (CDC), with the study protocol receiving approval from the NCHS Institutional Ethics Review Board. Informed written consent was obtained from all participants prior to their involvement in the survey.

**Figure 1:**
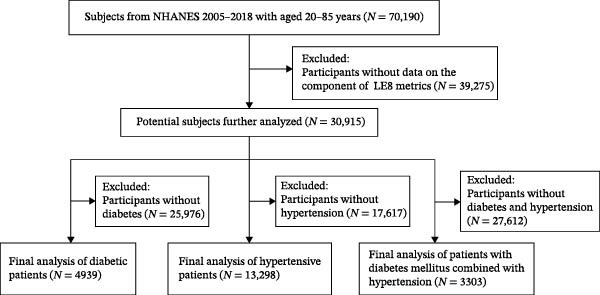
Flow chart of the sample collection in this study. LE8, life’s essential 8; NHANES, National Health and Nutrition Examination Survey.

Inclusion criteria: participants included in this study were required to meet the following criteria: (1) respondents aged 20 years or older from the 2005–2018 survey cycles (a total of seven cycles); (2) individuals with clear diagnoses of diabetes mellitus, hypertension, or concomitant diabetes mellitus and hypertension documented at the baseline survey; and (3) individuals with clearly defined follow‐up duration, survival status, cause of death, and health outcomes. Exclusion criteria: the study excluded participants based on the following criteria: (1) individuals with incomplete demographic information and (2) individuals missing data on LE8 scores. More details are provided in Figure 1.

### 2.2. Assessment of CVH by LE8

The LE8 scoring indicators consist of four health behaviors and four health factors related to diet, physical activity, tobacco/nicotine exposure, sleep health, BMI, non‐HDL cholesterol, blood glucose, and BP. Each indicator was scored on an ordinal scale ranging from 0 to 100 points. The total LE8 score was calculated based on the unweighted mean of the 8 indicators, and the AHA considers participants with LE8 scores between 80 and 100 as high CVH; 50–79 as moderate CVH; and 0–49 as low CVH [9]. In this study, we defined participants with LE8 scores of 0–49 as having low CVH and those with scores between 50 and 100 as having moderate‐to‐high CVH.

Dietary assessments were conducted using the 2015 Healthy Eating Index (HEI), where participants’ dietary intake data were collected through two 24 h dietary recalls [16]. These data were integrated with food pattern equivalents from the United States Department of Agriculture (USDA) to compute the HEI‐2015 scores [17]. Additionally, self‐reported questionnaires gathered information regarding physical activity levels, smoking status, sleep patterns, and diabetes history, including medication usage. Physical examinations were performed to measure BP, height, and weight, with BMI calculated as weight in kilograms divided by the square of height in meters. Blood samples were obtained and analyzed in a central laboratory to determine lipid profiles, plasma glucose levels, and hemoglobin A1c concentrations.

### 2.3. Diagnosis and Definitions

The diagnostic criteria for diabetes mellitus are established by the American Diabetes Association and include (1) glycosylated hemoglobin (HbA1c) levels of 6.5% or higher; (2) fasting plasma glucose (FPG) levels of 7 mmol/L or greater; (3) a 2 h plasma glucose level of 11.1 mmol/L or more during an oral glucose tolerance test; (4) random plasma glucose of 11.1 mmol/L or higher; (5) self‐reported history of diabetes mellitus; and (6) current use of insulin or other antidiabetic medications [18]. In accordance with guidelines set forth by the American College of Cardiology and AHA, hypertension is defined as having a systolic BP (SBP) of 140 mmHg or greater, a diastolic BP (DBP) of 90 mmHg or higher, a self‐reported history of hypertension, or previous use of antihypertensive medications [19].

### 2.4. Ascertainment of Mortality

Mortality data for adult participants in the NHANES were sourced from the National Death Index (NDI) as of December 31, 2019. The underlying causes of death were determined according to the International Classification of Diseases, 10^th^ edition (ICD‐10) definitions. All‐cause mortality was defined as any cause of death. Heart disease mortality was defined as ICD‐10 codes I00‐I09, I11, I13, and I20‐I51 [20]. Cancer mortality was defined as ICD‐10 codes C00‐C97 [21]. Follow‐up duration was defined as the time interval from the date of the interview to the date of death, or to December 31, 2019, for participants who remained event‐free.

### 2.5. Covariates

We selected age, sex, race/ethnicity, marital status, education, and the poverty income ratio as demographic variables. Alcohol consumption was also included as a variable for the lifestyle assessment. In this study, age was divided into two categories: ≤60 and >60 years. Race/ethnicity was categorized as non‐Hispanic White and other. Marital status was classified as married, unmarried, or divorced. Educational attainment was divided into less than high school and high school graduate or higher. The poverty income ratio was calculated by dividing family income by the poverty threshold and was divided into three groups: <1.3 (low income), 1.3–3.5 (middle income), and > 3.5 (high income) [22]. The participants’ current alcohol consumption status was categorized as “yes” or “no” depending on whether they had consumed more than 12 standard drinks in their lifetime.

### 2.6. Statistical Analyses

In light of the complex sampling design employed by the NHANES, all analyses in this study incorporated sample weights, clustering, and stratification to yield nationally representative estimates. Categorical variables were reported as counts and percentages. We utilized a weighted Cox proportional hazards model to investigate the associations between LE8 scores and the risks of all‐cause, heart disease, and cancer mortality among individuals diagnosed with diabetes, hypertension, and those with comorbid diabetes and hypertension. These associations are presented as hazard ratios (HRs) with corresponding 95% confidence intervals (CIs). The analysis comprised three distinct models: Model 1 served as the unadjusted model, Model 2 adjusted for gender, age, and race, while Model 3 further accounted for marital status, education level, income‐to‐poverty ratio, and alcohol consumption in addition to the covariates of Model 2. The proportional hazards assumption was evaluated using the Kaplan–Meier method, with survival probabilities estimated and visualized through weighted Kaplan–Meier curves. To explore potential nonlinear associations between LE8 scores and mortality outcomes, we employed restricted cubic splines (RCS) in the weighted Cox proportional hazards model. Furthermore, stratified analyses were conducted to assess whether the impact of LE8 scores on all‐cause, heart disease, and cancer mortality differed based on sex, age, race, marital status, educational attainment, poverty‐to‐income ratio, and alcohol consumption. Multiplicative interaction terms between strata, median LE8 scores, and outcomes were incorporated into the models to examine these interactions.

To investigate whether the association between LE8 scores and mortality is statistically consistent with mediation, we conducted formal statistical mediation analyses focusing on inflammatory biomarkers and biological aging. Specifically, we examined the NLR and PIV as markers of systemic inflammation, and PhenoAgeAccel as an indicator of biological aging. PhenoAgeAccel was calculated as the residual from regressing phenotypic age on chronological age, representing the deviation between an individual’s estimated biological age and their chronological age. Mediation analyses were performed using the “mediation” R package (version: 4.5.0). The proportion mediated was calculated as the ratio of the indirect effect to the total effect and expressed as a percentage. We used bootstrapping with 1000 replications to derive 95% CIs for the mediation estimates. All statistical analyses were conducted using R software (version 4.5.0). A two‐sided significance threshold of *p*  < 0.05 was employed to determine statistical significance.

## 3. Results

### 3.1. Baseline Characteristics of Participants

Our cohort included 4939 individuals with diabetes, 13,298 with hypertension, and 3303 with both conditions. Table 1 presents the detailed demographic and clinical characteristics stratified by mortality status. Bold values denote that the results are statistically significant. Across all three groups, decedents were significantly older and had lower poverty–income ratios compared to survivors (*p* < 0.05). In the diabetes cohort (median follow‐up: 73 months; IQR: 41–123), 940 deaths occurred, including 256 heart disease deaths. In the hypertension group (median follow‐up: 80 months; IQR: 44–124), 2425 deaths were recorded, with 637 from heart disease causes. Among those with both conditions (median follow‐up: 67 months; IQR: 38–113), 781 deaths occurred, including 213 heart disease deaths. As illustrated in Figure S1, LE8 scores exhibited marked differences across mortality outcomes in all patient cohorts, with significantly lower scores observed among deceased individuals compared to survivors.

**Table 1:** Demographic characteristics of the study population in NHANES 2005–2018.

Variables	Diabetes mellitus (*n* = 4939)			Hypertension (*n* = 13,298)			Diabetes mellitus and hypertension (*n* = 3303)		
	Surviving cases	All‐cause deaths	*p*	Surviving cases	All‐cause deaths	*p*	Surviving cases	All‐cause deaths	*p*
Sex, *n* (%)	—	—	**0.004**	—	—	0.643	—	—	0.885
Male	1646 (43.143)	527 (50.424)	—	5168 (48.502)	1318 (49.018)	—	1198 (48.839)	418 (48.408)	—
Female	2340 (56.857)	423 (49.576)	—	5690 (51.498)	1107 (50.982)	—	1323 (51.161)	363 (51.592)	—
Age (years), *n* (%)	—	—	**<0.001**	—	—	**<0.001**	—	—	**<0.001**
≤60	2155 (59.425)	168 (21.159)	—	5768 (60.080)	409 (21.407)	—	1020 (47.341)	134 (20.740)	—
>60	1831 (40.575)	782 (78.841)	—	5090 (39.920)	2016 (78.593)	—	1501 (52.659)	647 (79.260)	—
Ethnicity, *n* (%)	—	—	**<0.001**	—	—	**<0.001**	—	—	**<0.001**
Non‐Hispanic White	1328 (60.491)	477 (72.121)	—	4529 (69.500)	1490 (79.704)	—	818 (62.629)	390 (72.337)	—
Other	2658 (39.509)	473 (27.879)	—	6329 (30.500)	935 (20.296)	—	1703 (37.371)	391 (27.663)	—
Marital, *n* (%)	—	—	**<0.001**	—	—	**<0.001**	—	—	**<0.001**
Married	2323 (62.852)	449 (49.012)	—	5963 (60.504)	1111 (48.459)	—	1426 (62.020)	360 (48.814)	—
Single/separated	1659 (37.148)	500 (50.988)	—	4890 (39.496)	1313 (51.541)	—	1093 (37.980)	420 (51.186)	—
Education, *n* (%)	—	—	**<0.001**	—	—	**<0.001**	—	—	**<0.001**
Less than high school	1160 (19.029)	409 (34.639)	—	2633 (15.497)	863 (28.590)	—	773 (19.689)	332 (34.000)	—
High school or above	2821 (80.971)	539 (65.361)	—	8211 (84.503)	1561 (71.410)	—	1743 (80.311)	448 (66.000)	—
Poverty, *n* (%)	—	—	**<0.001**	—	—	**<0.001**	—	—	**<0.001**
<1.3	1213 (22.189)	339 (30.996)	—	2969 (19.183)	811 (28.146)	—	765 (22.401)	290 (31.778)	—
1.3–3.5	1447 (39.567)	403 (47.208)	—	3844 (36.929)	1016 (46.957)	—	941 (40.558)	319 (45.480)	—
>3.5	981 (38.244)	132 (21.796)	—	3108 (43.888)	420 (24.897)	—	589 (37.041)	111 (22.741)	—
Alcohol use, *n* (%)	—	—	**0.046**	—	—	**<0.001**	—	—	0.058
Yes	2922 (84.879)	726 (81.826)	—	8443 (88.832)	1909 (84.358)	—	1832 (85.056)	595 (81.584)	—
No	675 (15.121)	170 (18.174)	—	1468 (11.168)	380 (15.642)	—	418 (14.944)	146 (18.416)	—

*Note:* The bold values indicate that the differences between groups are statistically significant (*p* < 0.05).

### 3.2. Association Between LE8 Score and Mortality Risk

**Figure 2:**
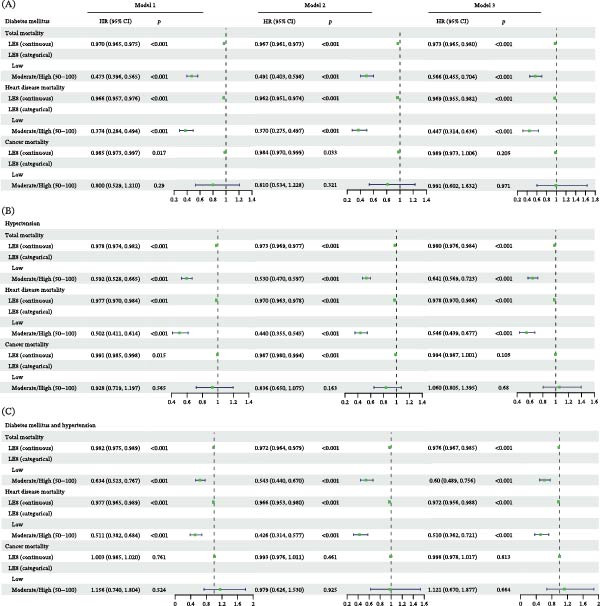
Forest plot illustrating the Cox proportional hazards regression analysis of the association between life’s essential 8 scores and mortality risk across three populations. Model 1 represents the unadjusted analysis. Model 2 adjusts for gender, age, and race. Model 3 further incorporates marital status, education level, income poverty status, and alcohol consumption in addition to the adjustments made in Model 2. (A) Diabetic population; (B) hypertensive population; (C) population with both diabetes and hypertension.

Higher LE8 scores were consistently associated with reduced mortality risk across all three patient populations (Figure 2). In fully adjusted models, each one‐point increase in the continuous LE8 score was associated with a 2.7% reduction in all‐cause mortality risk (HR = 0.973, 95% CI: 0.956–0.980) and a 3.1% reduction in heart disease mortality risk (HR = 0.969, 95% CI: 0.955–0.982) among individuals with diabetes. Similar protective associations were observed in individuals with hypertension and in those with both conditions. To address potential confounding by medication use, we conducted a sensitivity analysis by additionally adjusting Model 3 for antihypertensive, lipid‐lowering, and glucose‐lowering medications. The results were largely consistent with the primary Model 3 findings (Table S2), and the inverse associations between LE8 and both all‐cause and heart disease mortality persisted across all three patient groups.

When categorized, individuals with medium/high LE8 scores demonstrated substantially lower risks compared to those with low scores. Notably, the protective effects of higher LE8 scores were most pronounced for heart disease mortality across all three patient groups. While LE8 scores showed strong associations with all‐cause and heart disease mortality, no significant association was observed with cancer mortality after covariate adjustment.

### 3.3. Dose–Response Relationships and Survival Patterns

**Figure 3:**
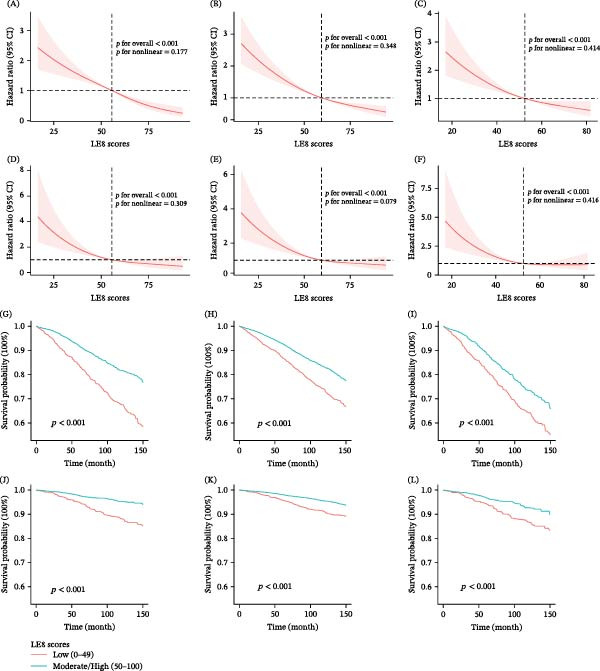
Restricted cubic spline plots depicting the association between life’s essential 8 (LE8) scores and mortality risk, and Kaplan–Meier survival curves depicting mortality risks stratified by LE8 score across different populations. (A) LE8 score and all‐cause mortality in diabetes. (B) LE8 score and all‐cause mortality in hypertension. (C) LE8 score and all‐cause mortality in diabetes‐hypertension comorbidity. (D) LE8 score and heart disease mortality in diabetes. (E) LE8 score and heart disease mortality in hypertension. (F) LE8 score and heart disease mortality in diabetes–hypertension comorbidity. (G) All‐cause mortality in diabetic individuals by LE8 score. (H) All‐cause mortality in hypertensive individuals by LE8 score. (I) All‐cause mortality in individuals with diabetes and hypertension by LE8 score. (J) Heart disease mortality in diabetic individuals by LE8 score. (K) Heart disease mortality in hypertensive individuals by LE8 score. (L) Heart disease mortality in individuals with diabetes and hypertension by LE8 score.

The RCS analyses revealed significant negative dose–response relationships between LE8 scores and both all‐cause and heart disease mortality risks (Figure 3). As LE8 scores increased, mortality risks decreased progressively across all three patient populations. No nonlinear relationships were observed between LE8 scores and cancer mortality (Figure S2). Kaplan–Meier survival analyses (Figure 3) demonstrated significantly better survival probabilities among individuals with medium/high LE8 scores compared to those with low scores for both all‐cause and heart disease mortality (log‐rank *p*  < 0.05).

### 3.4. Subgroup Analyses Reveal Differential Associations

Stratified analyses revealed important effect modifications by demographic and socioeconomic factors (Figure S5). For all‐cause mortality, the protective association of higher LE8 scores was particularly pronounced among younger individuals (≤60 years) with diabetes, hypertension, or both conditions; married individuals with diabetes or hypertension; higher‐income individuals with diabetes; and males with both conditions. For heart disease mortality, the protective effects of higher LE8 scores were dramatically stronger among younger individuals (≤60 years) with diabetes (HR = 0.156, 95% CI: 0.076–0.321), hypertension (HR = 0.681, 95% CI: 0.523–0.885), or both conditions (HR = 0.266, 95% CI: 0.120–0.589). For cancer mortality, associations were largely nonsignificant across subgroups.

### 3.5. Inflammation and Biological Aging Statistically Mediate LE8‐Mortality Associations

**Figure 4:**
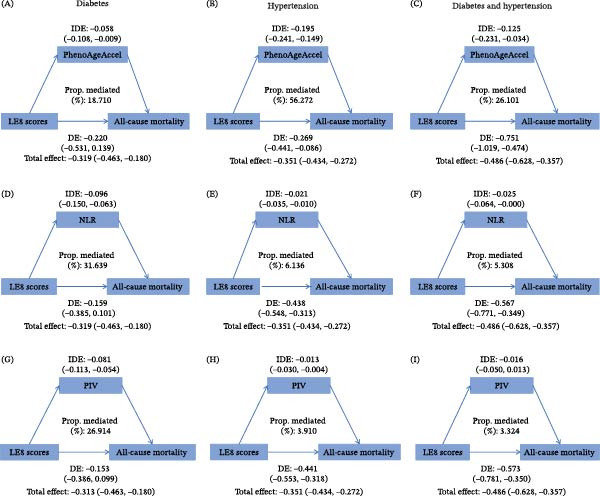
Statistical mediating effects of phenotypic age acceleration, neutrophil‐to‐lymphocyte ratio, and pan‐immune‐inflammation value on the association between LE8 scores and all‐cause mortality risk. (A, D, G) diabetic population, (B, E, H) hypertensive population, and (C, F, I) population with both diabetes and hypertension. DE, direct effect; IDE, indirect effect; NLR, neutrophil‐to‐lymphocyte ratio; PhenoAgeAccel, phenotypic age acceleration; PIV, pan‐immune–inflammation value.

Our mediation analyses revealed statistical associations consistent with mediation (Figure 4). In individuals with diabetes, the association between LE8 scores and all‐cause mortality showed statistically significant mediation by biological aging (PhenoAgeAccel: 18.7% of total effect) and by systemic inflammation markers (NLR: 31.6%; platelet‐to‐lymphocyte ratio [PIV]: 26.9%).

Among individuals with hypertension, PhenoAgeAccel accounted for the largest proportion of the statistical mediation (56.3%), while inflammatory markers showed smaller but still significant contributions (NLR: 6.1% and PIV: 3.9%). In participants with both conditions, PhenoAgeAccel (26.1%) and NLR (5.3%) significantly mediated the LE8‐mortality relationship, suggesting that both biological aging and inflammation may serve as key explanatory pathways linking CVH metrics to survival outcomes. Further analyses revealed similar mediating effects for heart disease mortality (Figure S7), particularly in individuals with hypertension or both conditions, where PhenoAgeAccel consistently emerged as the most significant mediator.

### 3.6. Sensitivity Analyses

The results of the sensitivity analyses further confirmed the stability of our primary conclusions. In the diabetes subgroup, the mediation effect of biological aging remained statistically significant even after employing the modified PhenoAgeAccel (excluding the glucose component), suggesting that the observed mediation was not a mathematical artifact of shared components (Figure S8). When further adjusting for the duration of diabetes and hypertension, the inverse association between higher LE8 scores and heart disease mortality remained consistent across all subgroups (Table S4).

Furthermore, the Fine‐Gray competing risk models yielded subdistribution HRs (SHRs) that were highly consistent with the primary Cox models, indicating that competing risks of death did not bias our estimates (Table S5 and Figure 2). Lastly, when the outcome was expanded to include stroke (broad CVD mortality), the protective association of high LE8 levels remained robust (Table S6), reinforcing the clinical relevance of our findings for hypertensive and diabetic populations.

## 4. Discussion

In this nationally representative cohort of US adults with diabetes, hypertension, or both conditions, we found that higher LE8 scores were significantly associated with reduced risks of all‐cause and heart disease mortality. Importantly, our mediation analyses suggested that inflammatory biomarkers and biological aging indices statistically explained a meaningful proportion of these associations, providing insight into potential pathways through which comprehensive CVH may relate to survival benefits.

Our findings indicate that inflammation showed the strongest statistical mediating association (31.6%) in individuals with diabetes, while PhenoAgeAccel demonstrated the largest proportion of association explained (56.3%) in hypertensive individuals. In those with both conditions, biological aging accounted for a notable proportion (26.1%), followed by inflammatory markers. These results align with growing evidence suggesting that chronic low‐grade inflammation contributes significantly to diabetic complications [11], while accelerated biological aging may be more central to hypertension‐related mortality risk [23]. Notably, these proportions should be interpreted as differences in statistical decomposition across mediator constructs rather than as definitive evidence that one biological process is absent in a given condition.

A key consideration is that diabetes is tightly linked to aging‐related biology, including advanced glycation end‐products (AGEs), oxidative stress, and “inflammaging” [24–27]. Thus, a larger proportion of the LE8‐mortality association explained by inflammatory indices in diabetes does not imply that biological aging is unimportant; rather, it may reflect differences in how alternative mediator constructs capture risk within disease‐defined populations. First, chronic hyperglycemia and AGE‐related tissue injury can amplify innate immune activation and endothelial dysfunction, which may be more directly reflected by inflammatory indices (e.g., NLR and PIV) and may also capture susceptibility to intercurrent infections or inflammatory stressors [28–32]. Second, PhenoAgeAccel is derived from a multibiomarker composite spanning metabolic, inflammatory/immune, renal, hepatic, and hematologic domains (e.g., albumin, creatinine, glucose, log‐CRP, lymphocyte percentage, MCV, RDW, alkaline phosphatase, and WBC) [15]. Within a diabetes‐defined subgroup, the incremental variability attributable to glycemia‐related aging processes may be partially constrained and/or statistically overlap with disease status and inflammatory signaling, whereas inflammatory indices may remain more heterogeneous across individuals, yielding a larger proportion of association explained. Third, in hypertension, cumulative vascular remodeling and end‐organ dysfunction may be more closely tracked by the multisystem physiological dysregulation embedded in PhenoAgeAccel, potentially resulting in a greater proportion of association explained [33, 34]. Taken together, these findings suggest that the observed differences are plausibly construct‐ and context‐dependent and should be interpreted cautiously as exploratory.

The protective associations between higher LE8 scores and reduced mortality risks can be explained through multiple pathways reflected in the LE8 components. Physical activity effectively modulates the sympathetic nervous system activity, improves endothelial function [35], and increases skeletal muscle sensitivity to insulin [36]. Exercise also favorably alters lipid profiles by decreasing triglycerides and LDL while elevating HDL levels [37]. We propose that these exercise‐induced effects may influence inflammatory and metabolic stress pathways, potentially contributing to the observed indirect associations through inflammatory markers in the diabetes subgroup.

Sleep health‐a novel addition to the LE8 framework‐appears particularly important in our findings. Sleep disturbances activate the autonomic nervous system and pro‐inflammatory transcription factors like NF‐κB, subsequently enhancing the expression of adhesion molecules and pro‐inflammatory mediators, ultimately leading to endothelial dysfunction [38–41]. The biological aging acceleration observed as a prominent indirect association in hypertension may partially reflect cumulative damage from sleep‐related disruptions in cellular homeostasis and neurohumoral regulation.

The differential patterns observed across disease groups suggest that the association between CVH and mortality risk may be connected through multiple overlapping biological processes. The relative proportions of association decomposition differed depending on the specific mediating constructs and clinical contexts. For diabetes, inflammatory biomarkers may more sensitively reflect immune–metabolic dysregulation and susceptibility to comorbid inflammatory/infectious stressors [31]; for hypertension, the multisystem physiological dysregulation summarized by PhenoAgeAccel may more effectively capture the cumulative long‐term burden [23]. Therefore, the present findings should be regarded as exploratory and hypothesis‐generating, rather than as conclusive evidence establishing a definitive hierarchy of disease‐specific mechanisms.

Our stratified analyses revealed important effect modifications by demographic and socioeconomic factors. The protective association of higher LE8 scores was particularly pronounced among younger individuals (≤60 years) across all three patient groups, suggesting that early lifestyle interventions may yield greater benefits. Similar enhanced protective effects were observed among those with higher socioeconomic status, potentially reflecting better access to resources supporting healthier lifestyles.

Previous studies have demonstrated that effective management of risk factors significantly impacts the prognosis in diabetic patients [42, 43]. The CARDIA study highlighted the potential benefits of CVH metrics in maintaining optimal CVH status in type 2 diabetes populations [44]. Our findings extend this knowledge by suggesting potential pathways—including inflammatory and biological aging‐related processes—through which CVH metrics may relate to mortality outcomes in these high‐risk populations.

Despite its strengths, including a large sample size, longitudinal design, and rigorous statistical analyses, this study has limitations. First, data on diet, physical activity, nicotine exposure, and sleep health rely on self‐reports, potentially introducing inaccuracies. Second, while we adjusted for numerous confounders, residual confounding cannot be completely eliminated, though E‐values (Table S1) suggest unmeasured confounding does not fully negate the observed associations. Third, since both the LE8 components and the mediator variables were measured at the same baseline time point, while mortality outcomes were ascertained prospectively, the concurrent assessment of the exposure and putative mediators prevents us from establishing temporal precedence. Consequently, our mediation results should be interpreted as identifying statistical associations consistent with a mediating model, rather than as evidence of a unidirectional causal cascade. Future studies with repeated measurements of LE8 components and mediator biomarkers are needed to confirm the temporal sequence and strengthen the causal inference. In addition, PhenoAgeAccel aggregates multiple biomarkers from domains such as metabolism, inflammation, and hematology, which makes it a comprehensive measure of biological aging. Our study was not designed to disentangle the influence of specific biomarkers or fully account for the potential confounding effects of subclinical infection or acute inflammatory states on inflammatory indices. Future studies should explore the contributions of individual biomarkers to PhenoAgeAccel and their relative roles in mediating mortality risk. Fourth, because LE8 and PhenoAge/PhenoAgeAccel partially share biomarker domains (notably glucose and CRP), we cannot fully exclude the possibility that a portion of the estimated indirect association reflects structural overlap and collinearity (mathematical coupling) rather than entirely distinct biological pathways; accordingly, we interpret the mediation proportions as exploratory and encourage future work to evaluate component‐level contributions and alternative, independently parameterized aging constructs. The findings of this study are most applicable to populations in which a complete LE8 score can be calculated. However, participants with complete versus incomplete LE8 data differed in baseline characteristics (Table S3). Therefore, the inability to include individuals with incomplete LE8 data may introduce some degree of selection bias, limiting the generalizability of our results to populations with missing LE8 components.

## 5. Conclusions

In conclusion, our study demonstrates that higher LE8 scores are associated with reduced mortality risk in individuals with diabetes, hypertension, or both conditions, with inflammatory and biological aging‐related processes potentially explaining part of these associations. These findings suggest that comprehensive CVH promotion may reduce mortality risk in these high‐risk populations partly through improvements in systemic inflammation and slower accumulation of multisystem physiological dysregulation. Future intervention and longitudinal studies should test whether targeted lifestyle programs can modify these biomarkers over time and whether such changes translate into improved survival.

**Nomenclature**
AHA: American Heart Association
BMI: Body mass index
BP: Blood pressure
CARDIA: Coronary artery risk development in young adults
CDC: Centers for disease control and prevention
CI: Confidence intervals
CVH: Cardiovascular health
CVD: Cardiovascular disease
DBP: Diastolic blood pressure
FPG: Fasting plasma glucose
HbA1c: Glycosylated hemoglobin
HDL: High‐density lipoprotein
HEI: Healthy eating index
HPA: Hypothalamic–pituitary–adrenal
HR: Hazard ratios
hsCRP: High‐sensitivity C‐reactive protein
ICD‐10: International Classification of Diseases, 10^th^ Edition
LDL: Low‐density lipoprotein
LE8: Life’s essential 8
LS7: Life’s simple 7
NCHS: National Center for Health Statistics
NDI: National Death Index
NF‐κB: Nuclear factor kappa B
NHANES: National Health and Nutrition Examination Survey
NLR: Neutrophil‐to‐lymphocyte ratio
PhenoAgeAccel: Phenotypic age acceleration
PIV: Pan‐immune inflammation value
RCS: Restricted cubic splines
SBP: Systolic blood pressure
USDA: United States Department of Agriculture.

## Author Contributions

**Xixi Dong and Xuejing Zhong:** writing – original draft, methodology, formal analysis. **Yichen Lin, Bingqin Xie, Xuefang Li, and Baochang He:** writing – review and editing. **Lingjun Yan and Fa Chen:** writing – review and editing, validation, methodology.

## Funding

This work was supported by the Joint Funds for the Innovation of Science and Technology of Fujian Province (Grant 2023Y9091), the Educational Research Program for Young and Middle‐aged Teachers in Fujian Province (Grant JZ230017), and the Technology Platform Construction Project of Fujian Province (Grant 2021Y2001).

## Ethics Statement

The NHANES data collection protocol was approved by the National Center for Health Statistics (NCHS) Research Ethics Review Board of the Centers for Disease Control and Prevention.

## Consent

All participants signed an informed consent form.

## Conflicts of Interest

The authors declare no conflicts of interest.

## Supporting Information

Additional supporting information can be found online in the Supporting Information section.

## Supporting information


**Supporting Information** Calculation of phenotypic age and phenotypic age acceleration (PhenoAgeAccel), and description of survey design and weighting. Table S1. *E* values for the association between life’s essential 8 and mortality risk among individuals with diabetes mellitus, hypertension, and their coexistence. Table S2. Association of life’s essential 8 (LE8) with all‐cause, heart disease, and cancer mortality after additional adjustment for medication. Table S3. Baseline characteristics of participants with complete versus missing life’s essential 8 (LE8) component data. Table S4. Association between life’s essential 8 (LE8) and heart disease mortality, further adjusted for disease duration. Table S5. Subdistribution hazard ratios (SHRs) for the association between LE8 and heart disease mortality using Fine‐Gray competing risk models. Table S6. Sensitivity analysis of the association between LE8 and cardiovascular disease (CVD) mortality across different adjustment models. Figure S1. Distribution of life’s essential 8 scores and mortality risks across different disease populations. Figure S2. Restricted cubic spline plots of life’s essential 8 (LE8) score and cancer mortality risk. Figure S3. Kaplan–Meier survival curves depicting cancer mortality risks stratified by life’s essential 8 (LE8) score across different populations. Figure S4. Forest plot illustrating the stratified analysis of the association between life’s essential 8 scores and all‐cause mortality risk across three populations. Stratification was performed by gender, age, race, marital status, education level, poverty–income ratio, and alcohol consumption status, with each stratum adjusted for the other variables mentioned. The low LE8 score group served as the reference group. Figure S5. Forest plot illustrating the stratified analysis of the association between life’s essential 8 scores and heart disease mortality risk across three populations. Stratification was performed by gender, age, race, marital status, education level, poverty–income ratio, and alcohol consumption status, with each stratum adjusted for the other variables mentioned. The low LE8 score group served as the reference group. Figure S6. Forest plot illustrating the stratified analysis of the association between life’s essential 8 scores and cancer mortality risk across three populations. Stratification was performed by gender, age, race, marital status, education level, poverty–income ratio, and alcohol consumption status, with each stratum adjusted for the other variables mentioned. The low LE8 score group served as the reference group. Figure S7. Statistical Mediating effects of phenotypic age acceleration, neutrophil‐to‐lymphocyte ratio, and pan‐immune‐inflammation value on the association between LE8 scores and heart disease mortality risk. Figure S8 Sensitivity analysis: statistical mediation of biological aging (Modified PhenoAge) on the association between LE8 and mortality in the diabetes subgroup.

## Data Availability

Data from the National Health and Nutrition Examination Survey (NHANES) 2005–2018 are publicly available online (https://www.cdc.gov/nchs/nhanes/index.htm).
