# Supplementary material for: Associations of Life’s Essential 8 With Mortality Among Individuals With Diabetes and/or Hypertension: Statistical Mediation by Inflammation and Biological Aging
Source: Mediators Inflamm. 2026 May 25;2026:3674573. doi: 10.1155/mi/3674573 (PMC13199995; doi:10.1155/mi/3674573)
Supplement: Supplementary file 1 — Supporting Information Calculation of phenotypic age and phenotypic age acceleration (PhenoAgeAccel), and description of survey design and weighting. Table S1. E values for the association between life’s essential 8 and mortality risk among individuals with diabetes mellitus, hypertension, and their coexistence. Table S2. Association of life’s essential 8 (LE8) with all‐cause, heart disease, and cancer mortality after additional adjustment for medication. Table S3. Baseline characteristics of participants with complete versus missing life’s essential 8 (LE8) component data. Table S4. Association between life’s essential 8 (LE8) and heart disease mortality, further adjusted for disease duration. Table S5. Subdistribution hazard ratios (SHRs) for the association between LE8 and heart disease mortality using Fine‐Gray competing risk models. Table S6. Sensitivity analysis of the association between LE8 and cardiovascular disease (CVD) mortality across different adjustment models. Figure S1. Distribution of life’s essential 8 scores and mortality risks across different disease populations. Figure S2. Restricted cubic spline plots of life’s essential 8 (LE8) score and cancer mortality risk. Figure S3. Kaplan–Meier survival curves depicting cancer mortality risks stratified by life’s essential 8 (LE8) score across different populations. Figure S4. Forest plot illustrating the stratified analysis of the association between life’s essential 8 scores and all‐cause mortality risk across three populations. Stratification was performed by gender, age, race, marital status, education level, poverty–income ratio, and alcohol consumption status, with each stratum adjusted for the other variables mentioned. The low LE8 score group served as the reference group. Figure S5. Forest plot illustrating the stratified analysis of the association between life’s essential 8 scores and heart disease mortality risk across three populations. Stratification was performed by gender, age, race, ma [file MI-2026-3674573-s001.docx]

**Supplementary methods**

**Phenotypic Age and Phenotypic Age Acceleration (PhenoAgeAccel).**

PhenoAgeAccel was previously developed and validated using National Health and Nutrition Examination Survey (NHANES) data and has been widely applied in population-based studies. Briefly, nine biomarkers—albumin, creatinine, glucose, C-reactive protein (CRP), lymphocyte percentage, mean corpuscular volume (MCV; NHANES: mean cell volume), red blood cell distribution width (RDW), alkaline phosphatase, and white blood cell (WBC) count—together with chronological age measured at baseline were used to compute Phenotypic Age (PhenoAge, in years). Following the established Gompertz-based parametrization, we first calculated the 10-year (120-month) mortality score as:

$$\mathrm{MortalityScor}e_{j}=1-\exp\left[ -\exp(xb_{j})\cdot\frac{\exp(\gamma t)-1}{\gamma} \right]$$

$$t=120\mathrm{months}, \gamma=0.0076927$$

The linear predictor $xb_{j}$ was computed as:

$$\begin{matrix} \mathrm{xb}_{j} & =-19.067-0.0336\cdot\mathrm{Albumin}_{j}+0.0095\cdot\mathrm{Creatinine}_{j}+0.1953\cdot\mathrm{Glucose}_{j} \\ & +0.0954\cdot\ln(\mathrm{CR}P_{j})-0.0120\cdot Lymphocyte{\%}_{j}+0.0268\cdot MCV_{j} \\ & +0.3306\cdot RDW_{j}+0.0019\cdot AlkalinePhosphatase_{j}+0.0554\cdot WBC_{j} \\ & +0.0804\cdot Age_{j} \end{matrix}$$

where biomarkers were entered in the following units: albumin (g/L), creatinine (µmol/L), glucose (mmol/L), CRP (mg/dL; natural log-transformed), lymphocyte percentage (%), MCV (fL), RDW (%), alkaline phosphatase (U/L), WBC count (10^3^ cells/µL), and age (years).

Next, PhenoAge (years) was derived by transforming the mortality score:

$$\mathrm{PhenoAge}_{j}=141.50225+\frac{\ln\left[ -0.0053\cdot\ln(1-\mathrm{MortalityScore}_{j}) \right]}{0.090165}$$

Finally, phenotypic age acceleration was defined using the residual-based approach, i.e., the residual from a linear regression of PhenoAge on chronological age:

$$\mathrm{PhenoAge}_{j}=\alpha+\beta\cdot\mathrm{Age}_{j}+\varepsilon_{j}$$

$$\quad\mathrm{PhenoAgeAccel}_{j}=\varepsilon_{j}$$

Positive PhenoAgeAccel values indicate that an individual’s phenotypic age is higher than expected for their chronological age (accelerated phenotypic aging), whereas negative values indicate a lower-than-expected phenotypic age.

**Survey design and weighting**

In light of the complex, multistage probability sampling design of NHANES, all analyses incorporated sampling weights and specified strata (SDMVSTRA) and primary sampling units (SDMVPSU) for design-based variance estimation. The present study pooled seven NHANES 2-year cycles (2005-2018); therefore, we constructed combined 14-year MEC examination weights by rescaling the 2-year MEC weight (WTMEC2YR) by the number of included cycles (WTMEC2YR/7) and applied these combined weights consistently in all survey-weighted analyses.

| **Supplementary Table 1** E values for the association between Life’s Essential 8 and mortality risk among individuals with diabetes mellitus, hypertension, and their coexistence. | | | | | |
| --- | --- | --- | --- | --- | --- |
| Variables | Model 1 |  | Model 2 |  | Model 3 |
|  | E-value (lower limit of 95% CI) |  | E-value (lower limit of 95% CI) |  | E-value (lower limit of 95% CI) |
| **Diabetes mellitus** |  |  |  |  |  |
| All-cause mortality |  |  |  |  |  |
| LE8 (continuous) | 1.17 (1.15) |  | 1.18 (1.16) |  | 1.16 (1.13) |
| LE8 (categorical) |  |  |  |  |  |
| Low | ref |  | ref |  | ref |
| Moderate/High (50-100) | 2.74 (2.33) |  | 2.65 (2.21) |  | 2.33 (1.87) |
| Heart disease mortality |  |  |  |  |  |
| LE8 (continuous) | 1.23 (1.18) |  | 1.24 (1.19) |  | 1.21 (1.15) |
| LE8 (categorical) |  |  |  |  |  |
| Low | ref |  | ref |  | ref |
| Moderate/High (50-100) | 4.79 (3.46) |  | 4.85 (3.44) |  | 3.90 (2.52) |
| Cancer mortality |  |  |  |  |  |
| LE8 (continuous) | 1.14 (1.06) |  | 1.14 (1.03) |  | 1.12 (1.00) |
| LE8 (categorical) |  |  |  |  |  |
| Low | ref |  | ref |  | ref |
| Moderate/High (50-100) | 1.81 (1.00) |  | 1.77 (1.00) |  | 1.10 (1.00) |
| **Hypertension** |  |  |  |  |  |
| All-cause mortality |  |  |  |  |  |
| LE8 (continuous) | 1.14 (1.13) |  | 1.16 (1.14) |  | 1.13 (1.12) |
| LE8 (categorical) |  |  |  |  |  |
| Low | ref |  | ref |  | ref |
| Moderate/High (50-100) | 2.23 (1.98) |  | 2.47 (2.21) |  | 2.06 (1.81) |
| Heart disease mortality |  |  |  |  |  |
| LE8 (continuous) | 1.18 (1.14) |  | 1.21 (1.17) |  | 1.17 (1.13) |
| LE8 (categorical) |  |  |  |  |  |
| Low | ref |  | ref |  | ref |
| Moderate/High (50-100) | 3.40 (2.64) |  | 3.97 (3.07) |  | 3.07 (2.32) |
| Cancer mortality |  |  |  |  |  |
| LE8 (continuous) | 1.10 (1.05) |  | 1.13 (3.07) |  | 1.08 (1.00) |
| LE8 (categorical) |  |  |  |  |  |
| Low | ref |  | ref |  | ref |
| Moderate/High (50-100) | 1.37 (1.00) |  | 1.68 (1.00) |  | 1.31 (1.00) |
| **Diabetes mellitus&hypertension** |  |  |  |  |  |
| All-cause mortality |  |  |  |  |  |
| LE8 (continuous) | 1.13 (1.10) |  | 1.16 (1.14) |  | 1.15 (1.11) |
| LE8 (categorical) |  |  |  |  |  |
| Low | ref |  | ref |  | ref |
| Moderate/High (50-100) | 2.08 (1.69) |  | 2.42 (1.97) |  | 2.17 (1.72) |
| Heart disease mortality |  |  |  |  |  |
| LE8 (continuous) | 1.18 (1.12) |  | 1.23 (1.16) |  | 1.20 (1.12) |
| LE8 (categorical) |  |  |  |  |  |
| Low | ref |  | ref |  | ref |
| Moderate/High (50-100) | 3.33 (2.28) |  | 4.13 (2.86) |  | 3.33 (2.12) |
| Cancer mortality |  |  |  |  |  |
| LE8 (continuous) | 1.06 (1.00) |  | 1.09 (1.00) |  | 1.05 (1.00) |
| LE8 (categorical) |  |  |  |  |  |
| Low | ref |  | ref |  | ref |
| Moderate/High (50-100) | 1.58 (1.00) |  | 1.17 (1.00) |  | 1.49 (1.00) |

| **Supplementary Table 2** Association of Life’s Essential 8 (LE8) with all-cause, heart disease, and cancer mortality after additional adjustment for medication | | | | | | | | |
| --- | --- | --- | --- | --- | --- | --- | --- | --- |
| **Outcome** | **Diabetes mellitus** | |  | **Hypertension** | |  | **Diabetes & Hypertension** | |
| **All-cause mortality** | HR (95% CI) | *P* |  | HR (95% CI) | *P* |  | HR (95% CI) | *P* |
| LE8 (continuous) | 0.975 (0.967, 0.983) | <0.001 |  | 0.981 (0.977, 0.985) | <0.001 |  | 0.977 (0.968, 0.986) | <0.001 |
| LE8 (categorical) |  |  |  |  |  |  |  |  |
| Low | ref | ref |  | ref | ref |  | ref | ref |
| Moderate/High | 0.599 (0.480, 0.747) | <0.001 |  | 0.655 (0.579, 0.741) | <0.001 |  | 0.513 (0.364, 0.722) | <0.001 |
| **Heart disease mortality** |  |  |  |  |  |  |  |  |
| LE8 (continuous) | 0.971 (0.957, 0.986) | <0.001 |  | 0.980 (0.972, 0.987) | <0.001 |  | 0.972 (0.957, 0.988) | <0.001 |
| LE8 (categorical) |  |  |  |  |  |  |  |  |
| Low | ref | ref |  | ref | ref |  | ref | ref |
| Moderate/High | 0.476 (0.334, 0.679) | <0.001 |  | 0.564 (0.454, 0.702) | <0.001 |  | 1.128 (0.675, 1.887) | 0.645 |
| **Cancer mortality** |  |  |  |  |  |  |  |  |
| LE8 (continuous) | 0.992 (0.975, 1.009) | 0.332 |  | 0.995 (0.988, 1.003) | 0.203 |  | 0.998 (0.979, 1.018) | 0.835 |
| LE8 (categorical) |  |  |  |  |  |  |  |  |
| Low | ref | ref |  | ref | ref |  | ref | ref |
| Moderate/High | 1.043 (0.636, 1.713) | 0.866 |  | 1.073 (0.813, 1.417) | 0.618 |  | 0.610 (0.491, 0.758) | <0.001 |
| Note: Adjusted for sex, age, race/ethnicity, marital status, education level, poverty-to-income ratio, alcohol consumption, and baseline medication use (antihypertensive, lipid-lowering, and glucose-lowering medications). | | | | | | | | |

| **Supplementary Table 3** Baseline characteristics of participants with complete versus missing Life’s Essential 8 (LE8) component data | | | | |
| --- | --- | --- | --- | --- |
| Variables | Total  (n=70190) | Complete LE8  (n=30915) | Missing LE8  (n=39275) | *P* |
| Sex, n (%) |  |  |  | < 0.001 |
| Female | 35,481 (50.550) | 16,205 (52.418) | 19,276 (49.080) |  |
| Male | 34,709 (49.450) | 14,710 (47.582) | 19,999 (50.920) |  |
| Age (years), n (%) |  |  |  | 0.1094 |
| > 60 | 12,627 (17.990) | 9,883 (31.968) | 2,744 (6.987) |  |
| ≤ 60 | 27,122 (38.641) | 21,032 (68.032) | 6,090 (15.506) |  |
| Ethnicity, n (%) |  |  |  | < 0.001 |
| Non-Hispanic White | 25,326 (36.082) | 13,591 (43.962) | 11,735 (29.879) |  |
| Other | 44,864 (63.918) | 17,324 (56.038) | 27,540 (70.121) |  |
| Marital, n (%) |  |  |  | < 0.001 |
| Married | 20,284 (28.899) | 16,205 (52.418) | 4,079 (10.386) |  |
| Single/separated | 21,148 (30.130) | 14,696 (47.537) | 6,452 (16.428) |  |
| Education, n (%) |  |  |  | < 0.001 |
| High school | 10,062 (14.335) | 7,115 (23.015) | 2,947 (7.504) |  |
| Less than high school | 27,373 (38.998) | 7,193 (23.267) | 20,180 (51.381) |  |
| Some college or above | 21,131 (30.105) | 16,579 (53.628) | 4,552 (11.590) |  |
| Poverty, n (%) |  |  |  | < 0.001 |
| < 1.3 | 24,042 (34.253) | 8,647 (27.970) | 15,395 (39.198) |  |
| 1.3-3.5 | 23,367 (33.291) | 10,887 (35.216) | 12,480 (31.776) |  |
| > 3.5 | 16,449 (23.435) | 8,886 (28.743) | 7,563 (19.257) |  |
| Alcohol use, n (%) |  |  |  | < 0.001 |
| No | 5,355 (7.629) | 4,019 (13.000) | 1,336 (3.402) |  |
| Yes | 30,236 (43.077) | 25,271 (81.743) | 4,965 (12.642) |  |

| **Supplementary Table 4** Association between Life’s Essential 8 (LE8) and Heart Disease Mortality, further adjusted for disease duration | | |
| --- | --- | --- |
| **Outcome** | **HR (95% CI)** | ***P*** |
| **Diabetes mellitus** |  |  |
| **Heart disease mortality** |  |  |
| LE8 (categorical) |  |  |
| Low | ref | ref |
| Moderate/High | 0.464 (0.322, 0.668) | <0.001 |
| **Hypertension** |  |  |
| **Heart disease mortality** |  |  |
| LE8 (categorical) |  |  |
| Low | ref | ref |
| Moderate/High | 0.551 (0.408, 0.745) | <0.001 |
| **Diabetes & Hypertension** |  |  |
| **Heart disease mortality** |  |  |
| LE8 (categorical) |  |  |
| Low | ref | ref |
| Moderate/High | 0.444 (0.276, 0.714) | <0.01 |

Note: Models were adjusted for age, sex, ethnicity, education level, marital status, poverty-to-income ratio, alcohol use, and duration of diabetes or hypertension.

| **Supplementary Table 5** Sub-distribution Hazard Ratios (SHR) for the association between LE8 and heart disease mortality using Fine-Gray competing risk models | | |
| --- | --- | --- |
| **Outcome** | **Fine-Gray SHR (95% CI)** | ***P*** |
| **Diabetes mellitus** |  |  |
| **Heart disease mortality** |  |  |
| LE8 (categorical) |  |  |
| Low | ref | ref |
| Moderate/High | 0.544 (0.418, 0.708) | <0.001 |
| **Hypertension** |  |  |
| **Heart disease mortality** |  |  |
| LE8 (categorical) |  |  |
| Low | ref | ref |
| Moderate/High | 0.692 (0.577, 0.831) | <0.001 |
| **Diabetes & Hypertension** |  |  |
| **Heart disease mortality** |  |  |
| LE8 (categorical) |  |  |
| Low | ref | ref |
| Moderate/High | 0.626 (0.470, 0.834) | <0.01 |

Note: Adjusted for sex, age, race/ethnicity, marital status, education level, poverty-to-income ratio, and alcohol consumption.

| **Supplementary Table 6** Sensitivity analysis of the association between LE8 and cardiovascular disease (CVD) mortality across different adjustment models. | | | | | | | | |
| --- | --- | --- | --- | --- | --- | --- | --- | --- |
| **Outcome** | **Model 1** | |  | **Model 2** | |  | **Model 3** | |
| **Diabetes mellitus** |  |  |  |  |  |  |  |  |
| **CVD mortality** | HR (95% CI) | *P* |  | HR (95% CI) | *P* |  | HR (95% CI) | *P* |
| LE8 (continuous) | 0.963 (0.954, 0.973) | <0.001 |  | 0.959 (0.948, 0.970) | <0.001 |  | 0.966 (0.952, 0.981) | <0.001 |
| LE8 (categorical) |  |  |  |  |  |  |  |  |
| Low | ref | ref |  | ref | ref |  | ref | ref |
| Moderate/High | 0.358 (0.281, 0.456) | <0.001 |  | 0.371 (0.287, 0.480) | <0.001 |  | 0.443 (0.325, 0.603) | <0.001 |
| **Hypertension** |  |  |  |  |  |  |  |  |
| **CVD mortality** |  |  |  |  |  |  |  |  |
| LE8 (continuous) | 0.974 (0.968, 0.980) | <0.001 |  | 0.967 (0.961, 0.974) | <0.001 |  | 0.975 (0.968, 0.983) | <0.001 |
| LE8 (categorical) |  |  |  |  |  |  |  |  |
| Low | ref | ref |  | ref | ref |  | ref | ref |
| Moderate/High | 0.490 (0.416, 0.578) | <0.001 |  | 0.430 (0.362, 0.511) | <0.001 |  | 0.534 (0.443, 0.643) | <0.001 |
| **Diabetes & Hypertension** |  |  |  |  |  |  |  |  |
| **CVD mortality** |  |  |  |  |  |  |  |  |
| LE8 (continuous) | 0.973 (0.960, 0.986) | <0.001 |  | 0.962 (0.948, 0.976) | 0.203 |  | 0.967 (0.950, 0.985) | <0.001 |
| LE8 (categorical) |  |  |  |  |  |  |  |  |
| Low | ref | ref |  | ref | ref |  | ref | ref |
| Moderate/High | 0.484 (0.373, 0.628) | <0.001 |  | 0.418 (0.318, 0.550) | <0.001 |  | 0.484(0.356,0.658) | <0.001 |

Note: Model 1 represents the unadjusted analysis. Model 2 adjusts for gender, age, and race. Model 3 further incorporates marital status, education level, income poverty status, and alcohol consumption in addition to the adjustments made in Model 2.


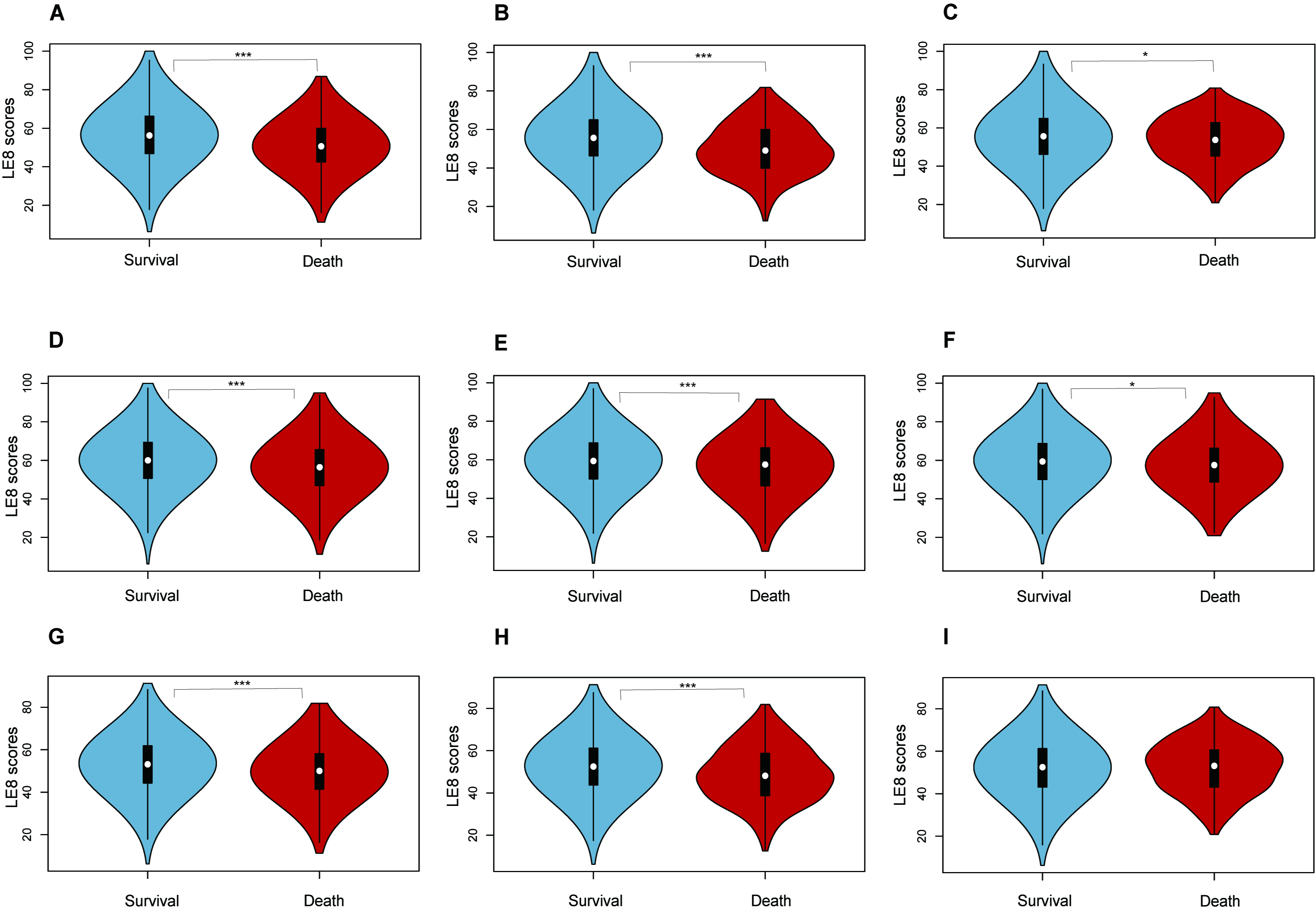


**Supplementary Figure 1** Distribution of life’s essential 8 scores and mortality risks across different disease populations. **(A)** For all-cause mortality in the diabetic population; **(B)** For heart disease mortality in a diabetic population; **(C)** For cancer mortality in the diabetic population; **(D)** For all-cause mortality in the hypertensive population; **(E)** For heart disease mortality in a hypertensive population; **(F)** For cancer mortality in the hypertensive population; **(G)** For all-cause mortality in individuals with the two diseases co-occurrence; **(H)** For heart disease mortality in individuals with the two diseases co-occurrence; **(I)** For cancer mortality in individuals with the two diseases co-occurrence.


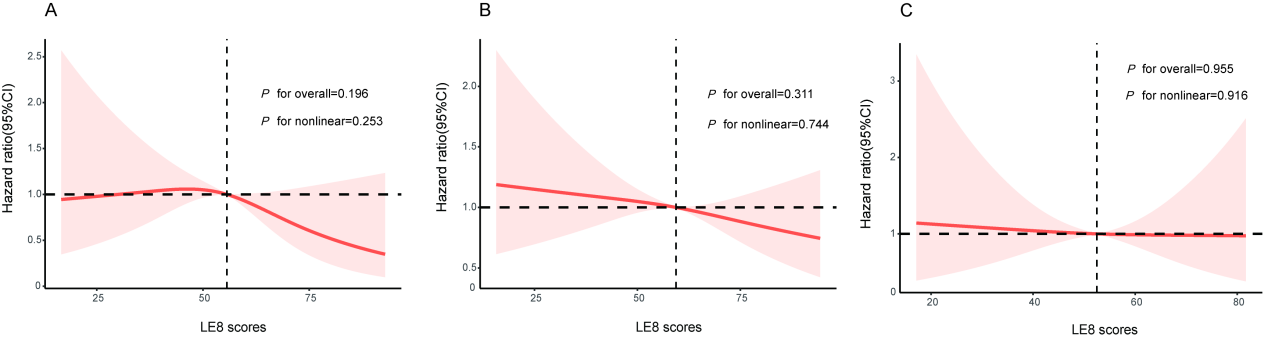


**Supplementary Figure 2** Restricted cubic spline plots of life’s essential 8 (LE8) score and cancer mortality risk. **(A)** LE8 score and cancer mortality in diabetes; **(B)** LE8 score and cancer mortality in hypertension; **(C)** LE8 score and cancer mortality in diabetes-hypertension comorbidity.


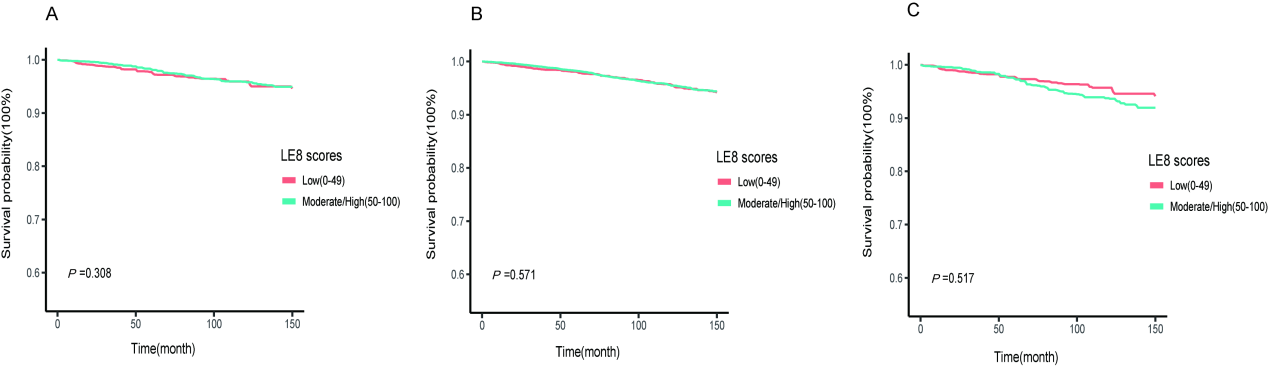


**Supplementary Figure 3** Kaplan-Meier survival curves depicting cancer mortality risks stratified by life’s essential 8 (LE8) score across different populations. **(A)** cancer mortality in diabetic individuals by LE8 score; **(B)** cancer mortality in hypertensive individuals by LE8 score; **(C)** cancer mortality in individuals with diabetes and hypertension by LE8 score.


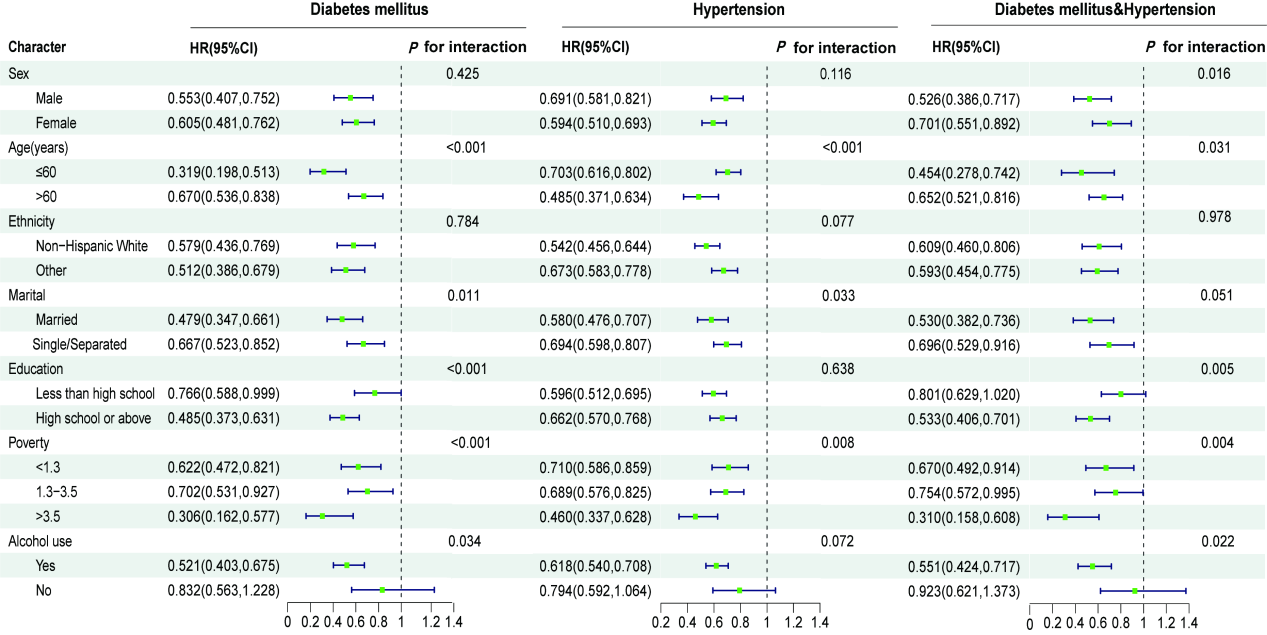


**Supplementary Figure 4** Forest plot illustrating the stratified analysis of the association between life’s essential 8 scores and all-cause mortality risk across three populations. Stratification was performed by gender, age, race, marital status, education level, poverty-income ratio, and alcohol consumption status, with each stratum adjusted for the other variables mentioned. The low LE8 score group served as the reference group.


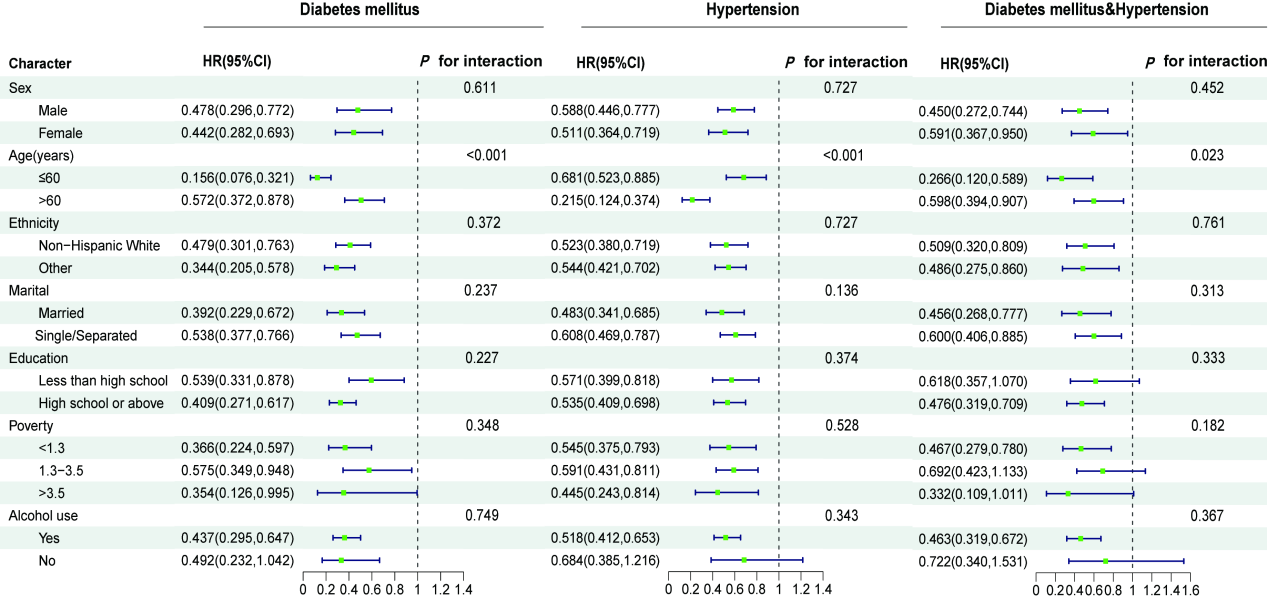


**Supplementary Figure 5** Forest plot illustrating the stratified analysis of the association between life’s essential 8 scores and heart disease mortality risk across three populations. Stratification was performed by gender, age, race, marital status, education level, poverty-income ratio, and alcohol consumption status, with each stratum adjusted for the other variables mentioned. The low LE8 score group served as the reference group.


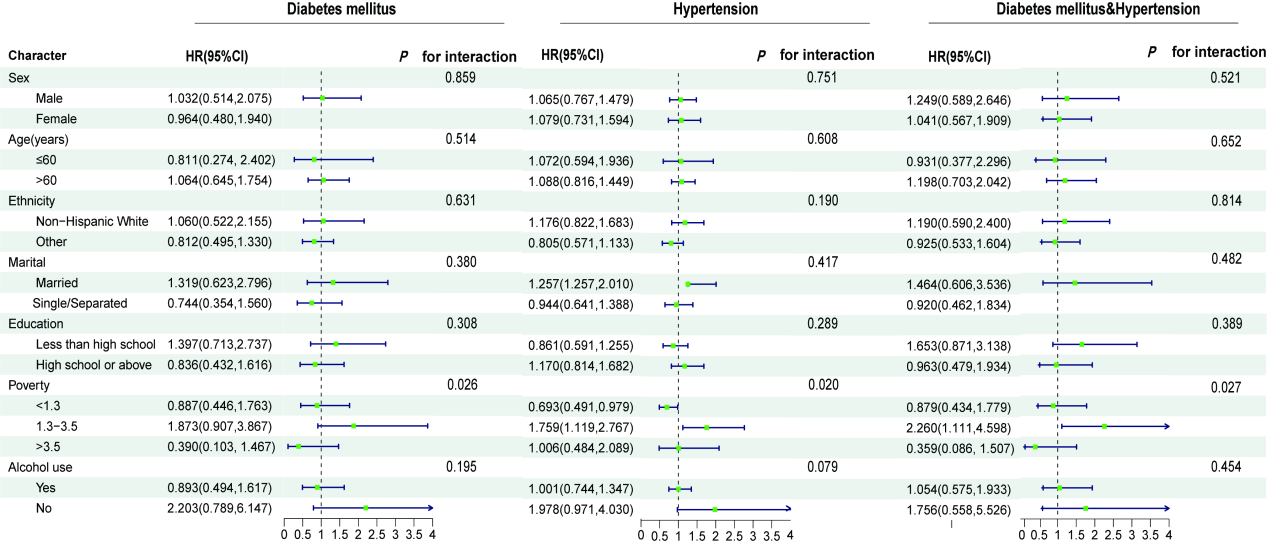


**Supplementary Figure 6** Forest plot illustrating the stratified analysis of the association between life’s essential 8 scores and cancer mortality risk across three populations. Stratification was performed by gender, age, race, marital status, education level, poverty-income ratio, and alcohol consumption status, with each stratum adjusted for the other variables mentioned. The low LE8 score group served as the reference group.


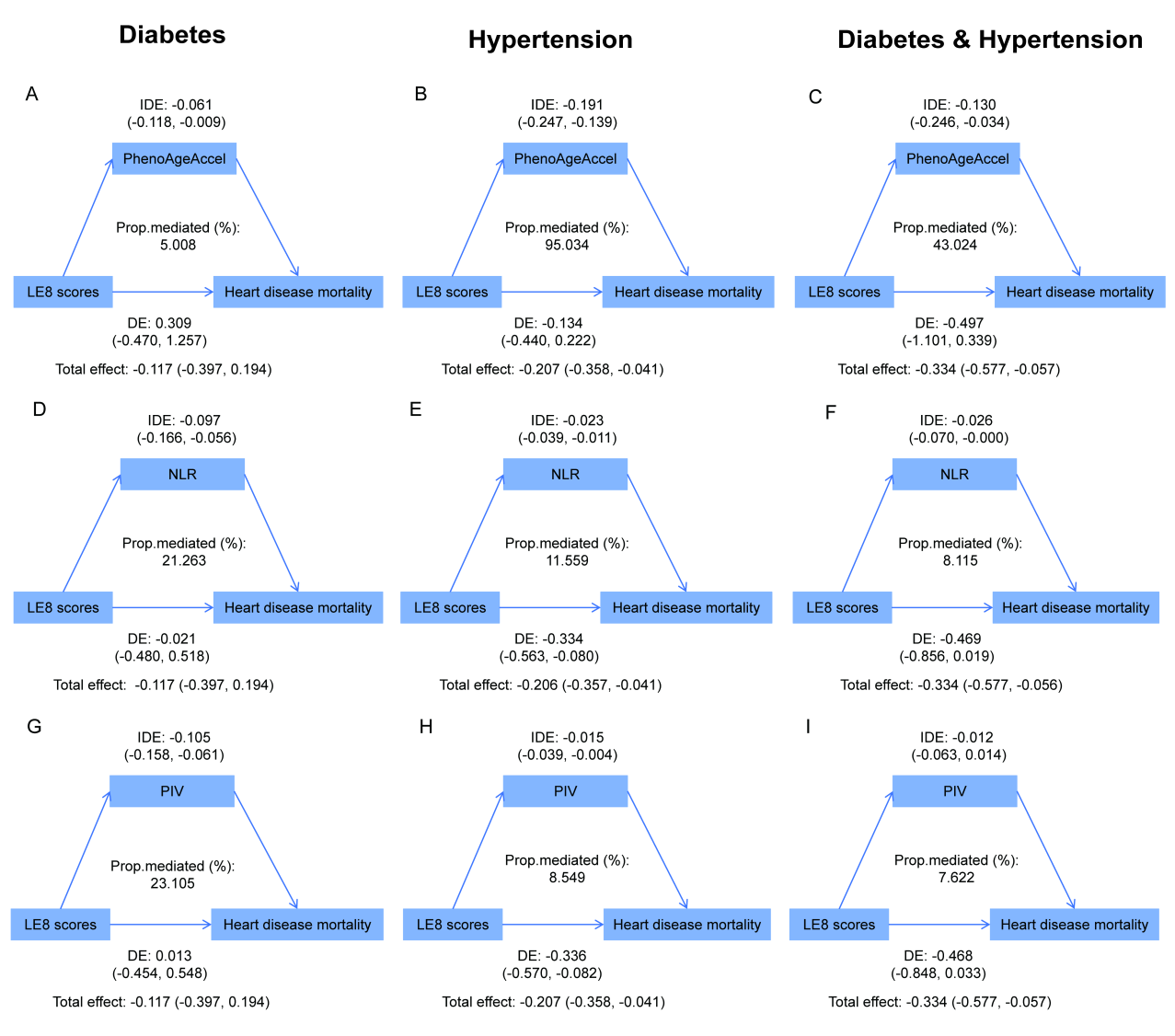


**Supplementary Figure 7** Statistical Mediating effects of phenotypic age acceleration, neutrophil-to-lymphocyte ratio, and pan-immune-inflammation value on the association between LE8 scores and heart disease mortality risk. **(A, D, G)**: diabetic population; **(B, E, H)**: hypertensive population; **(C, F, I)**: population with both diabetes and hypertension. Abbreviation: DE, direct effect; IDE, indirect effect; NLR, neutrophil-to-lymphocyte ratio; PhenoAgeAccel, phenotypic age acceleration; PIV, pan-immune-inflammation value.


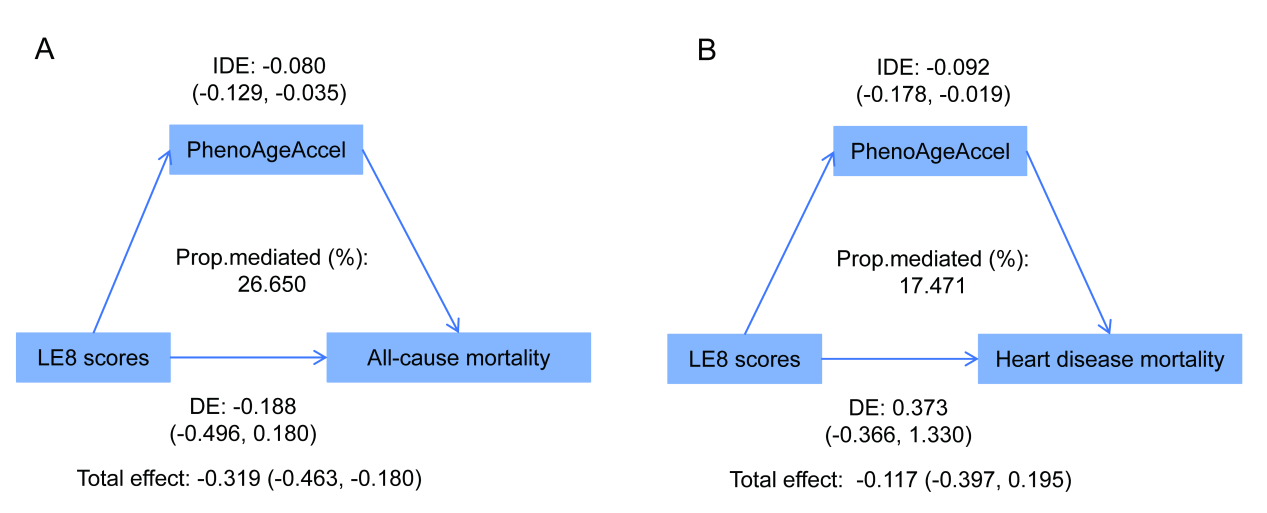


**Supplementary Figure 8** Sensitivity Analysis: Statistical Mediation of Biological Aging (Modified PhenoAge) on the Association Between LE8 and Mortality in the Diabetes Subgroup. **(A)** For all-cause mortality in the diabetic population; **(B)** For heart disease mortality in a diabetic population.
